# Challenges to Improve Bone Healing Under Diabetic Conditions

**DOI:** 10.3389/fendo.2022.861878

**Published:** 2022-03-28

**Authors:** Yiling Chen, Yue Zhou, Jie Lin, Shiwen Zhang

**Affiliations:** ^1^ State Key Laboratory of Oral Diseases and National Clinical Research Center for Oral Diseases, West China Hospital of Stomatology, Sichuan University, Chengdu, China; ^2^ Department of Oral Implantology, West China Hospital of Stomatology, Sichuan University, Chengdu, China

**Keywords:** diabetic bone disease, bone healing, hyperglycemia, mesenchymal stem cells, biomedical cues, diabetic drug

## Abstract

Diabetes mellitus (DM) can affect bone metabolism and the bone microenvironment, resulting in impaired bone healing. The mechanisms include oxidative stress, inflammation, the production of advanced glycation end products (AGEs), etc. Improving bone healing in diabetic patients has important clinical significance in promoting fracture healing and improving bone integration. In this paper, we reviewed the methods of improving bone healing under diabetic conditions, including drug therapy, biochemical cues, hyperbaric oxygen, ultrasound, laser and pulsed electromagnetic fields, although most studies are in preclinical stages. Meanwhile, we also pointed out some shortcomings and challenges, hoping to provide a potential therapeutic strategy for accelerating bone healing in patients with diabetes.

## Introduction

**Figure 1:**
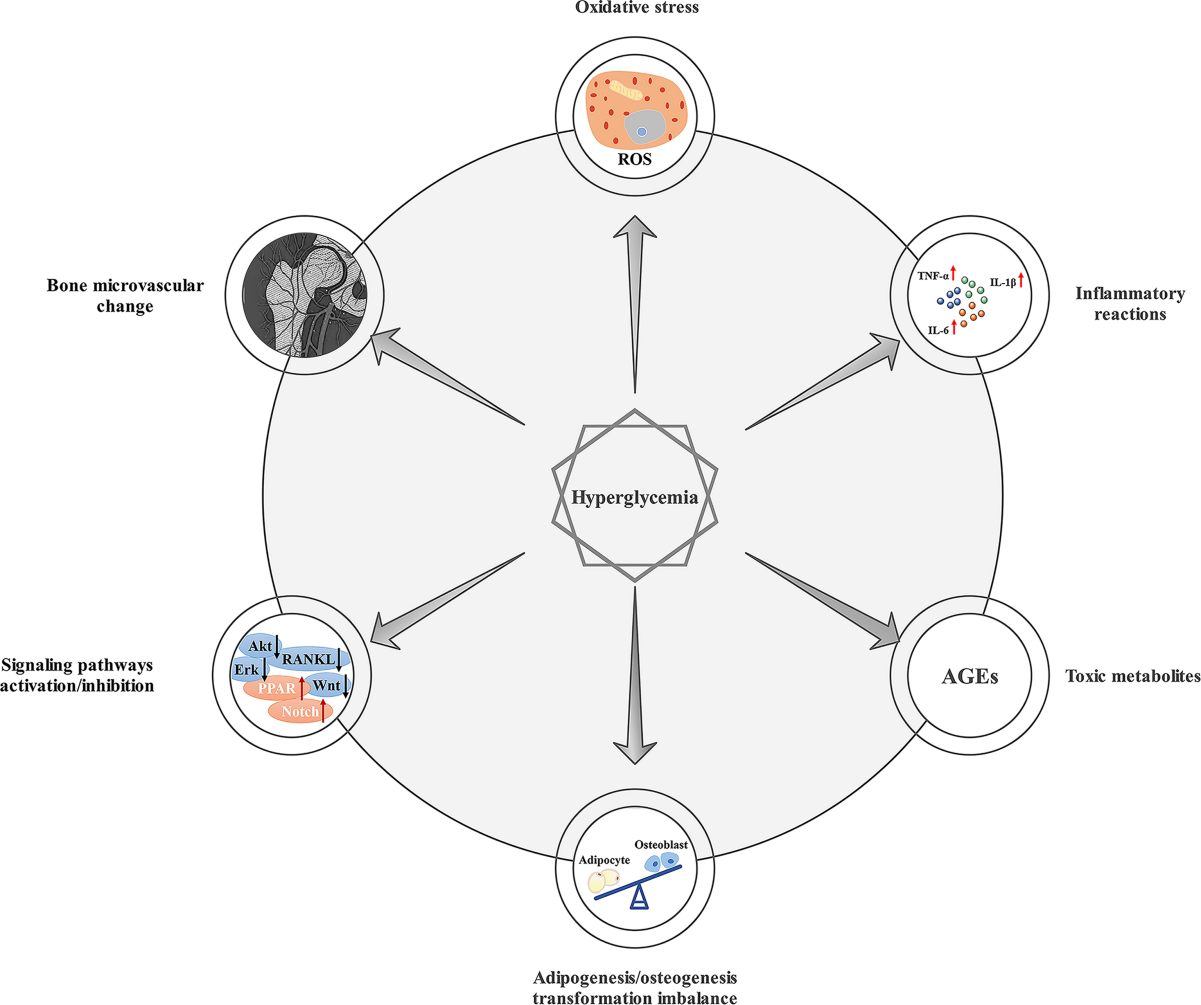
Mechanisms of chronic hyperglycemia on bone healing. Chronic hyperglycemia can lead to oxidative stress, inflammatory reactions, adipogenesis/osteogenesis transformation imbalance, the production of AGEs, signaling pathway activation/inhibition, and bone microvascular changes, which can result in impaired bone healing.

Diabetes is a metabolic disorder caused by insufficient insulin secretion and/or insulin resistance in target tissue (1). In 2019, approximately 463 million people worldwide had diabetes, with an adult prevalence rate of 9.3%. The adult prevalence of diabetes is expected to reach 10.2% by 2030 (2). Numerous clinical and *in vivo* or *in vitro* studies have shown that type 1 diabetes mellitus (T1DM) and type 2 diabetes mellitus (T2DM) both increase the risk of fractures and have a negative impact on bone healing (3). Compromised bone healing in patients with diabetes is associated with changes in the interaction between osteoblasts, adipocytes, bone marrow stem cells (BMSCs), and the marrow environment (4). Generally, chronic hyperglycemia can lead to oxidative stress, inflammatory reactions, adipogenesis/osteogenesis transformation imbalance, signaling pathway activation/inhibition, and bone microvascular changes (5). The premature accumulation of senescent cells may also lead to accelerated bone aging (6), which results in reduced bone strength and impaired bone formation. And it is worth mentioning that hyperglycemia has been linked to the formation of AGEs, which play a primordial role in damaged bone healing in DM (7), and may increase the risk of fracture in T1DM (8) and T2DM (9). As an important part of inflammatory events in diabetes and its complications, AGEs, interacting with the receptor for AGEs (RAGE), participates in the development of diabetic osteopathy (10). Mechanisms of chronic hyperglycemia on bone healing are summarized in **Figure 1**. However, the mechanisms leading to compromised bone healing in T1DM and T2DM are different. In T1DM, reduced insulin synthesis and amylin deficiency may initiate cascading reactions (11). In T2DM, compromised bone healing may be attributed to insulin resistance and impaired incretin effects (12). At present, bone healing in diabetic states is mainly improved by hypoglycemic therapy (13). Nevertheless, many other treatments have been shown in laboratory studies to promote bone healing in diabetic states, which may become potential strategies in further clinical studies. Therefore, this paper reviews methods for improving bone healing under diabetic conditions, only mechanisms of drugs or therapies possibly associated with benefits will be reviewed, hoping to provide potential strategies for accelerating bone healing in diabetic states.

## Drugs

### Anti-Diabetes Drugs

Patients with diabetes need to take oral or injectable anti-diabetes drugs to control blood glucose. Studies have suggested that longer disease duration and worse glycemic control is associated with higher fracture risks in patients with diabetes (14). At the same time, anti-diabetes drugs may directly influence bone cells or indirectly affect bone metabolism and bone healing. Metformin is the first-line drug for the treatment of diabetes recognized worldwide and is widely used in all stages of diabetes treatment. Metformin can increase the intracellular AMP/ATP ratio, thereby activating the AMP-activated protein kinase (AMPK) signaling pathway, triggering the rapid activation of insulin and lysosomes, reducing intracellular glucose levels, and ultimately promoting the proliferation and differentiation of osteoblasts (15). The direct effect of metformin on osteogenesis functions by activating the BMP-4/Smad/Runx2 signaling pathway, upregulating the expression of Runt-related transcription factor 2 (Runx2) (16), activating extracellular signal-regulated kinases (ERK) 1/2 and p42/p44 mitogen-activated protein kinase (MAPK) (17), inhibiting the GSK-3β/Wnt/β-catenin pathway to promote the osteogenic differentiation of human BMSCs (18), and significantly increasing the formation of MSC mineralized nodules through the LKB1/AMPK pathway (19). A large number of experiments have confirmed that metformin can directly affect bone formation through the joint regulation of the multiple signaling pathways mentioned above. On the other hand, metformin can reduce intracellular reactive oxygen species (ROS) accumulation and inhibit AGEs-induced cellular inflammation through AMPK activation and RAGE/NF-κB pathway, thereby reducing oxidative stress and indirectly affecting bone healing (20–22). In terms of implants, experiments have proven that metformin can significantly promote the formation of new bone, improve the microstructure of bone, and promote the osseointegration of implants in both direct and indirect ways, such as increasing the autophagy of osteoporotic BMSCs under hypoxic and serum deprivation culture conditions, reducing ROS production and increasing the expression of osteogenic markers (23). This shows that metformin also has potential application prospects in the application of oral implants.

Intestinal hormones can accelerate glucose absorption by inducing the pancreas to secrete insulin, including glucose-dependent insulinotropic peptide (GIP) and glucagon-like peptide-1 (GLP-1). On the one hand, GIP can inhibit the apoptosis of osteoblasts and BMSCs (24, 25). On the other hand, GIP reduces the activity of osteoclasts in a dose-dependent manner and inhibits bone resorption induced by parathyroid hormone (PTH) and receptor activator of nuclear factor-κB ligand (RANKL) (26).

The level of GLP-1 rises immediately after a meal, while the circulating level of intact GLP-1 decreases rapidly under dipeptidyl peptidase-4 (DPP-4)-mediated inactivation. Therefore, GLP-1 receptor agonists and DPP-4 inhibitors are effective drugs for treating T2DM (27). GLP-1 receptor agonists can increase the serum level of the bone formation marker osteocalcin, thereby increasing bone mass and strength (28). Both exendin-4 and liraglutide are GLP-1 receptor agonists that can not only promote bone formation directly but also inhibit osteoclast differentiation and bone resorption. Exendin-4 has been shown to promote the differentiation of BMSCs into osteoblasts through the Wnt/β-catenin signaling pathway (29). At the same time, experiments have demonstrated that liraglutide can activate the PI3K/AKT, ERK1/2, and cAMP/PKA/β-cat-Ser675 signaling pathways through GLP-1 receptors and directly promote osteogenic proliferation and differentiation in the osteoblast cell line MC3T3-E1 (30). On the other hand, studies have found that Exendin-4 can inhibit RANKL-induced osteoclast differentiation and bone resorption by activating GLP-1 receptors, thereby preventing bone trabecular microstructure deterioration and enhancing bone strength (31). It can also inhibit macrophages to produce tumor necrosis factor-α (TNF-α) in lipopolysaccharide (LPS) environments, thereby reducing LPS-induced osteoclast differentiation and bone resorption (32).

DPP-4 inhibitors, including sitagliptin, saxagliptin and linagliptin, achieve blood glucose control in patients with diabetes by extending the circulating half-life period of endogenous insulin. Animal experiments have shown that the DPP-4 inhibitor linagliptin can inhibit the increase in TNF-α and RANKL mRNA expression levels in mice treated with LPS, prevent LPS-induced osteoclast differentiation and bone resorption, induce macrophages to differentiate to the M2 phenotype, and inhibit TNF-α-induced osteoclast differentiation and dental root resorption, thereby improving inflammation-induced bone resorption (33). There are relatively few human studies on the effects of DPP-4 inhibitors on bones. However, meta-analysis and cohort studies have shown that DPP-4 inhibitors can reduce the risk of fractures compared with placebo and other anti-diabetic drugs (34–36).

Studies on the effects of sulfonylureas on bone metabolism are still lacking, and there are conflicting results in existing data. Some studies have shown that it has a potential stimulating impact on the proliferation and differentiation of osteoblasts and has a protective effect on osteoblasts in hyperglycemic environments (5, 37), but some studies have found that it increases the risk of falls and fractures (38, 39). Therefore, further research is needed on the effect of sulfonylureas on bone metabolism.

The effect of anti-diabetes drugs on bone healing under diabetic conditions should be fully considered in clinical application. In the absence of other contraindications or adverse reactions, doctors should minimize the use of drugs such as thiazolidinediones that may lead to diabetes bone-related complications (40) and try to use metformin, DPP-4I, GLP-1 receptor agonists and other drugs that can effectively promote bone healing to improve the overall bone condition of patients with diabetes.

### Natural Medicine Extracts

In recent years, natural medicine extracts have received increasing attention. Studies have found that several natural medicine extracts play an active role in bone regeneration. The following is a brief introduction to natural medicine extracts that can improve bone healing. Berberine, curcumin and resveratrol all play a dual role in antidiabetes and promoting bone healing and have broad clinical application prospects. Studies have found that berberine can activate osteoblast and osteoblast biomarker genes and promote bone regeneration. It can also inhibit bone loss caused by the T2DM drug pioglitazone through the AMPK pathway (41). The latest study by Jingjing Shao et al. (42) found that berberine may upregulate the ROS-mediated IRS-1 signaling pathway, thereby reducing the osteogenesis of BMSCs inhibited by high glucose. In addition, *in vivo* studies have shown that the combined application of berberine and insulin can promote implant osseointegration in diabetic rats (43). Curcumin is a polyphenolic phytochemical that is mainly extracted from turmeric. It has a positive effect on preventing and treating osteoporosis and bone deterioration in rheumatoid arthritis and other inflammatory diseases (44). In improving diabetic bone healing, curcumin has been shown to inhibit bone resorption and reduce the number of osteoclasts stimulated by diabetes (45). Studies in recent years have further confirmed that curcumin can promote bone repair around titanium implants in diabetic rats induced by streptozotocin through the Wnt/β-catenin signaling pathway and upregulation of BMP-2 levels (46). Resveratrol is a polyphenol plant antitoxin that is mainly extracted from plants and fruits (such as grapevines). Research by Yunwei Hua et al. (47) showed that resveratrol, as a Sirtuin1 agonist, can activate Sirtuin1, inhibit sclerostin, and reduce the impact of diabetes on implant osseointegration, resulting in increased bone density, improved trabecular bone structure, and biomechanical fixation enhancement. Moreover, in a double-blind randomized controlled trial in 2018, 192 T2DM patients were randomized to resveratrol 500 mg/day (Resv500), Resv40 mg/day (Resv40), or placebo. The results showed that resveratrol is effective in preventing bone density loss in patients with T2DM (48).

In addition, there are natural medicine extracts such as baicalein, ophiopogonin D (OP-D), and tubeimoside I (TBMS1). Although they do not have anti-diabetic effects, they have also been proven in preclinical studies to improve bone healing in diabetic animal models. These drugs still lack more experiments to verify their effectiveness in clinical applications. *In vivo* experiments of baicalein have been proven to have no effect on the insulin sensitivity index in chronic periodontitis with DM animal models, but baicalein can reduce alveolar bone loss in diabetic rats by increasing the expression of phospho-nuclear factor erythroid 2-related factor 2 (pNrf2) (49). Increased expression of pNrf2 can mediate the Nrf2/Keap1 signaling pathway to maintain the proliferation, migration, pluripotency and regeneration ability of MSCs in diabetic rats and ultimately promote bone healing in diabetic models (50). OP-D can act as an antioxidant to protect cells from oxidative stress-induced damage (51). Recent studies have found that OP-D inhibits oxidative stress through a Wnt/β-catenin-dependent mechanism, thereby reducing the damaged osseointegration of titanium implants under diabetic conditions (52). TBMS1 is a pentacyclic triterpene saponin compound isolated from the plant Fritillaria Vulgaris. *In vivo* and *in vitro* experiments targeting osteoclasts and RANKL-induced signaling pathways have found that TBMS1 has a protective effect on bone loss in T2DM rats by inhibiting osteoclast formation, bone resorption, and the RANKL-induced NF-κB signaling pathway (53).

Morroniside is mainly extracted from Cornus officinalis. A study by Yi Sun et al. (54) found that morroniside can inhibit the AGE-RAGE signaling system by activating Glo1, thereby restoring the osteogenic differentiation of rat BMSCs exposed to hyperglycemic conditions. *In vivo* experiments in a T1DM rat model also showed that morroniside can reduce bone loss and improve bone microstructure. Glycyrrhizin is a high mobility group box-1 (HMGB1) inhibitor. Experiments have shown that glycyrrhizin can significantly attenuate the upregulation and interaction of HG-induced ligand receptors for RAGE by inhibiting HMGB1, reducing oxidative stress, reversing the downregulation of osteogenic markers, and promoting osteogenic differentiation (55).

Traditional Chinese natural medicine and extracts of natural medicine are often used under diabetic conditions as a supplement rather than professional medical advice. While natural medicine extracts are popular, there are still regulation, safety and efficacy concerns. Except for resveratrol, none of the other natural medicinal extracts listed above has been tested in clinical trials for bone healing in diabetic patients. The preclinical trials of natural medicinal extracts in diabetic bone healing have not been examined thoroughly, and the mechanism of natural medicinal extracts in improving diabetic bone healing is not completely clear. At the same time, there is still a lack of clinical trials. Although we believe that natural medicine extracts have great application prospects and are highly likely to become an important complementary therapy for diabetic bone healing, a large number of reproducible experiments are still needed to confirm the efficacy and safety of natural medicinal extracts in promoting diabetic bone healing.

### Osteoporosis Drugs

Effective anti-osteoporosis drugs, such as vitamin D, bisphosphonates (e.g., risedronate, zoledronate and alendronate), sclerotin antibody, selective estrogen receptor modulators (e.g., raloxifene and bazedoxifene) and monoclonal RANKL antibodies, have also been shown to play a positive role in promoting bone healing under diabetic conditions. Vitamin D is an essential steroid hormone for the human body, and 1α,25-dihydroxy vitamin D3 (1,25VD3), as the main active form of vitamin D3, binds to the vitamin D binding protein in the plasma to reach target tissues and exert endocrine effects, regulating the metabolism of calcium and phosphorus. Experiments have shown that 1,25VD3 can reverse the reduction in osseointegration and mechanical strength, reduce the damage caused by AGEs-induced osteogenic differentiation, and downregulate the expression of RAGE, thereby promoting the osseointegration of T2DM titanium implants (56). A recent longitudinal study in 35 postmenopausal women found that bone fragility due to T2DM and aging was significantly improved after 1000 IU daily vitamin D supplementation for 12 months (57). In an *in vivo* study, risedronate reduced the number and function of osteoclasts in diabetic mice, and increased bone mineral density and vertebral mechanical strength in the femoral shaft and vertebral body (58). In another experiment, both alendronate and raloxifene were found to have an anti-bone resorption effect in diabetic animal models, reducing bone turnover rate and increasing bone mechanical strength (59). Zoledronate and sclerotin antibodies also prevented bone defects by reversing the adverse effects of diabetes on bone mass and strength in rats (60, 61). At the same time, there are clinical trials demonstrating the effectiveness of these drugs in the treatment of diabetic osteoporosis patients. A retrospective analysis of the 3-year placebo-controlled freedom study and the 7-year extension study found that denosumab significantly increased bone mineral density and reduced the risk of vertebral fracture in patients with osteoporosis and diabetes (62). Another retrospective national cohort study in Denmark found that patients treated with alendronate and denosumab had a similar risk of osteoporotic fractures in patients with diabetes, suggesting that the two agents have similar efficacy in preventing osteoporotic fractures in patients with diabetes (63). In exploratory trials of postmenopausal women with T2DM, treatment with either bazedoxifene or raloxifene improved bone resorption markers without affecting glucose metabolism (64, 65). Therefore, we believe that these anti-osteoporosis drugs have a potential role in promoting bone healing in diabetic patients, which may provide new ideas for the treatment of diabetic osteoporosis or poor bone healing. Finding drugs that can effectively control blood glucose while maintaining bone health may become a direction for future research in the treatment of diabetic osteoporosis.

### Other Drugs

In addition to the abovementioned anti-diabetes drugs, natural medicine extracts and osteoporosis drugs, some drugs also have a significant effect on promoting bone healing in patients with diabetes. Doxycycline is a tetracyclic antibiotic that is often used clinically to treat various infectious diseases caused by sensitive gram-positive bacteria and gram-negative bacilli. *In vitro* experiments have found that doxycycline may restore the vitality and proliferation of osteoblasts and the osteogenesis process of BMSCs under diabetic conditions through the Wnt/β-catenin signaling pathway (66). In addition, zinc is considered to be a potential drug to prevent bone loss caused by diabetes. Studies have found that both zinc carbonate and zinc sulfate have a positive effect on maintaining bone structure and biomechanical parameters (67). Among them, zinc sulfate can prevent diabetic osteoporosis, and its protective mechanism is mainly related to its hypoglycemic effect, inhibiting bone marrow adipogenesis and upregulating the OPG/RANKL ratio and RUNX2 (68). At present, relevant clinical studies have reported that zinc supplements have achieved obvious efficacy in the treatment of postmenopausal women with osteoporosis, but unfortunately, there is currently a lack of clinical trials and reports on the application of doxycycline and zinc supplements in the treatment of compromised bone healing under diabetic conditions. We believe that both doxycycline and zinc supplements can be used as complementary treatments for compromised diabetic bone healing. Doxycycline is especially suitable for the nonantibacterial treatment of patients with systemic inflammation, while zinc supplements are suitable for promoting diabetic bone healing by reducing the RANK/OPG ratio.

Moreover, previous studies have shown that diabetic complications also play an important role in causing bone fragility and damaging bone health in patients with diabetes. A study concerning aging individuals with T1DM for more than 50 years indicated that lipid profile is associated with bone mineral density (BMD) values, suggesting that drugs controlling cardiovascular disease may be useful in promoting bone health in T1DM (69). Additionally, diabetic microvascular complications including neuropathy, retinopathy, and nephropathy are relevant to increased fracture risks (70, 71). Therefore, drugs that can prevent or improve diabetic microvascular complications such as aspirin, angiotensin-converting enzyme inhibitors, statins may be of potential benefits to bone health under diabetic conditions (72).

## Biochemical Cues

### Hormones

Previous studies have revealed that various hormones can promote bone healing under diabetic conditions. Uncarboxylated osteocalcin is a multifunctional hormone secreted by mature osteoblasts (73) that plays an essential role in promoting the osteogenic differentiation of BMSCs (74). In hyperglycemic environments, uncarboxylated osteocalcin can inhibit adipogenic differentiation and promote osteogenic differentiation of BMSCs, the mechanism of which is to reduce the expression of TP63 and then affect the PTEN/Akt/GSK3β signaling pathway (75). Adrenomedullin 2 (ADM2) is an endogenous peptide belonging to the calcitonin family (76) that decreases significantly under diabetic conditions, and the reduction in ADM2 levels is associated with DM-related metabolic disorders (77). Studies have shown that ADM2 therapy can promote M1 macrophage polarization toward the M2 phenotype by activating the PPARγ-mediated NF-κB signaling pathway and improve bone regeneration in diabetic rats (78). Norepinephrine (NE) is the primary medium of the sympathetic nervous system, and its ability to affect MSC migration has been shown in previous experiments both *in vitro* and *in vivo (* 79). A study comparing BMSCs in diabetic mice undergoing sympathectomy with those that did not experience sympathectomy found that NE may have a protective effect on hyperglycemia-induced MSC apoptosis through the AKT/BCL-2 pathway (80). PTH enhances the MSC survival rate by inhibiting their aging and apoptosis (81) and promotes MSC differentiation toward osteogenesis rather than adipogenesis (82). In the fight against glucolipotoxicity, PTH can promote osteogenic differentiation of BMSCs by activating the p38 MAPK signaling pathway (83). Moreover, *in vivo* studies have shown that human parathyroid hormone (1–34) has a positive effect on bone fracture healing in T2DM (84). Recombinant human parathyroid hormone has been used as a drug to cure osteoporosis clinically (85). However, clinical trials are needed to further assess the effectiveness of recombinant hPTH in bone healing under diabetic conditions. Adiponectin (APN) plays a vital role in regulating energy metabolism at the cellular and systemic levels in diabetic environments (86). In patients with T2DM, APN concentration in plasma was significantly reduced, which is associated with damage in bone healing in diabetic states (87). In addition, APN activates the AMPK signaling pathway, plays an antioxidant role and protects mitochondria, reverses osteoblast damage, and improves bone integration in titanium implants (88). Therefore, integrating APN in scaffold-based systems may serve as a potential strategy to promote bone healing, but it still needs further investigation.

### Signaling Pathway Regulators

Many studies have demonstrated that activating or inhibiting signaling pathways associated with BMSC osteogenesis by designing specific signaling pathway regulators can promote bone healing in diabetic environments. GSK-3β, a kinase involved in blood glucose regulation (89), is a negative regulator of the Wnt signaling pathway and plays an important role in the regulation of bone metabolism (90). In hyperglycemic environments, GSK-3β is activated, and the Wnt signaling pathway is inhibited, damaging the proliferation of BMSCs (91). CHIR99021 is a GSK-3β inhibitor that inhibits the expression of β-catenin and CyclinD1 in hyperglycemic environments, promoting osteogenesis of BMSCs (92). In addition, previous studies have found that LiCl at 15 mM, as an inhibitor of GSK-3β, can also effectively reverse the inhibitory effect of hyperglycemia on BMSC osteogenesis (93). Brain and muscle ARNT-like protein 1 (BMAL1) is a core biological clock protein secreted by the suprachiasmatic nucleus, peripheral tissue, and stem cells (94). The study found that the overexpression of BMAL1 restores the bone-forming ability of BMSCs from diabetic rats by inhibiting the expression of the NF-κB signaling pathway (95). Therefore, bone metabolic balance in T2DM can be reconstructed by overexpression of BMAL1. As a glycoprotein secreted on the surface of cells, Semaphorin3B is closely related to the bone metabolism process, and it can improve defects in BMSC proliferation and osteogenesis in hyperglycemic environments by activating the Akt signaling pathway (96). Growth differentiation factor 11 (GDF11) is a bone morphological protein (BMP) whose expression is positively correlated with the incidence of osteoporosis in diabetic patients (97). A study was conducted to regularly inject GDF11 inhibitors into the tooth extraction socket in T2DM pigs, and it found that GDF11 can improve bone healing in the tooth extraction socket and promote osteogenesis of MSCs under T2DM conditions (98). Although these signaling pathway regulators mentioned above are only currently used in laboratories, they help us understand the underlying mechanisms in compromised bone healing under diabetic conditions, and they can be used as potential therapeutic targets to recover abnormal bone homeostasis caused by DM.

### Growth Factors

BMP is one of the most potent inducers of bone differentiation in MSCs (99). In the BMP family, BMP2, BMP4, BMP6, BMP7 and BMP9 all have bone-forming properties (100). Studies in streptozocin-induced diabetic mice have shown that BMP6 treatment can reduce bone loss in diabetic mice and that BMP6 plays an important role in T1DM-related bone loss (101). Vascular endothelial growth factors (VEGF) induce bone formation through direct and indirect pathways. VEGF can directly attract MSCs and promote osteogenesis while also promoting local angiogenesis, enhancing vascular permeability, accumulating MSCs, and indirectly promoting bone regeneration (102). Basic fibroblast growth factor (bFGF) is a mitogen that regulates bone cell proliferation, differentiation and mineralization (103). Several studies have shown a synergistic effect on promoting bone differentiation of MSCs between BMP and VEGF (100) and between BMP, VEGF and bFGF (104). This joint application is a new and promising improvement strategy in bone tissue engineering. In future studies, researchers can further explore the factors affecting its synergy and effectively promote bone regeneration in diabetic environments. Insulin-like growth factors (IGF1), when combined with IGF1 receptors, play an essential role in bone development, growth and physiological strength maintenance but require daily injections or surgical implants. A current study has shown that expressing codon-optimized Pro-IGF-1 with e-peptide in the chloroplast can be administered orally, significantly promoting bone regeneration in diabetic mice (105). This new type of administration can not only facilitate affordability but also enhance patient compliance; therefore, it may be a potential treatment for bone healing in diabetic patients. Progranulin (PGRN) is a multifunctional cytokine that has been shown to promote cartilage formation and participate in physiological fracture healing mainly through TNF receptor 2 (TNFR2) signaling pathways (106). The latest experiments have found that local application of recombinant PGRN in diabetic rats can effectively promote the healing of diabetic fractures. The mechanism of promoting cartilage formation may be related to the TNFR2-Akt and ERK1/2 pathways, as well as its role in inhibiting inflammation in the process of diabetic bone regeneration (107, 108).

The combination of bone-induced growth factors with bone-conductive biomaterials is also an important and promising way to promote bone regeneration (109). Studies have shown that implanting 3D bioprint scaffolds containing MSNs/BMP-4, BMSCs and RAW264.7 in bone defects can significantly promote bone healing in diabetic rats, and the mechanism is that BMP-4 can directly promote BMSC bone formation. BMP-4 can regulate macrophage cell differentiation to M2 macrophage polarization to improve the inflammatory microenvironment (110). Jian Li et al. (111) developed a BMP-2 and VEGF-derived peptide-decorated n-HA/PA66 (BVHP66) scaffold that significantly enhances the proliferation of BMSCs and human umbilical vein endothelial cells (HUVECs), promotes bone differentiation of BMSCs and blood vessel formation in hyperglycemic environments, and therefore improves bone healing in diabetic environments. In addition, some studies have achieved the delivery of growth factors through nonviral gene therapy, which can avoid the side effects of overdosing on recombinant human proteins (112). Behnoush Khorsand et al. (113) prepared a composite scaffold by building plasmid DNA encoding BMP-2 and FGF-2 combined with polyethylenimine through electrostatic action, which has been shown to improve bone regeneration in diabetic rabbits. In complicated diabetic environments, an autonomous tissue engineering system that can release growth factors accurately according to the dynamic environment is required in future research. Moreover, clinical experiments are also expected to be evaluated.

### Exosomes

Exosomes, with a diameter of 50-100 nm, are extracellular organelles secreted by cells and are capable of carrying bioactive substances such as noncoding RNA, mRNA, DNA, proteins, and other molecules (114). Exosomes can transport specific miRNAs and mediate cell and tissue-to-tissue communication (115). Studies have shown that both exosomes secreted by bone marrow stem cells in rats with T1DM (dBMSC-exos) and exosomes secreted by normal rat bone marrow stem cells (nBMSC-exos) can promote bone regeneration and angiogenesis, but the effect of dBMSC-exos is weaker than that of nBMSC-exos (116). Studies have also shown that exosomes derived from adipose-derived mesenchymal stem cells (AMSCs) inhibit the secretion of IL-1β and IL-18 by osteoclasts in hyperglycemic environments, reducing bone absorption and restoring bone loss (117). However, at present, MSC exosome-related research is still in the preclinical stage, and the traditional methods of exosome separation and characterization identification are not effective for clinical application. In the future, we need to further explore the exact mechanism of MSC exosomes in bone formation, bone cell differentiation, angiogenesis, inflammatory response, etc. Developing large-scale methods for the production, separation and purification of exosomes is also of vital importance.

Several miRNAs regulate BMSC proliferation, migration, differentiation and apoptosis and are important regulatory factors in bone healing (118). The miRNA from the exosome source can be steadily transferred from the bone microenvironment to BMSCs, regulating bone differentiation and bone healing, but the hyperglycemic environment can affect its expression. The study found that in hyperglycemic environments, the expression of miR-124-3p in osteocyte-derived exosomes may inhibit the expression of Gal-3 in osteoblasts, which in turn can lead to a decrease in bone capacity (119). MiR-144-5p levels also rise in exosomes secreted by diabetic bone marrow-derived macrophages, which can be transferred to BMSCs to inhibit bone regeneration by targeting Smad1 (120). Designing specific inhibitors of the miRNA as mentioned above may be a potential strategy to reverse the adverse effects of hyperglycemia on bone healing in diabetic patients.

## Physical Therapies

### Hyperbaric Oxygen

Hyperbaric oxygen (HBO) treatment refers to intermittent inhalation of 100% oxygen at pressures above 1.5 absolute atmospheric pressures (121). Hyperbaric oxygen therapy can improve the biometric properties of the femur in diabetic animal models and increase the content of collagen and crystalline hydroxyapatite (122). In addition, some studies have evaluated the effectiveness of hyperbaric oxygen therapy for implant bone integration in diabetic states through diabetic animal models, and the results showed that HBO therapy can enhance implant bone integration by calculating BIC through tissue morphology (123). However, some studies suggest that HBO only improves early bone integration in diabetic rabbits, which is not enough to improve the mechanical stability of implants (124). Therefore, it can be determined that HBO has positive effects on bone integration in diabetic environments at the histological and biomechanical levels, but there is a lack of studies of its specific mechanisms, and further clinical research is needed to evaluate the effectiveness of HBO as an auxiliary treatment for diabetic patients.

### Ultrasound

Ultrasound is an oscillating longitudinal pressure wave with a frequency over 20 kHz that cannot be detected by the human auditory system. Ultrasound has applications in many medical fields, including low-intensity pulsed ultrasound (LIPUS) for diagnostic imaging, medium-intensity ultrasound for physical therapy, and high-intensity focused ultrasound for surgical resection. LIPUS has been used clinically for more than 20 years. A large number of *in vivo* and *in vitro* experiments and clinical trials have verified the safety and effectiveness of LIPUS in promoting bone healing (125, 126). However, there are relatively few studies on LIPUS in bone healing in patients with diabetes. Past experiments have shown that in diabetic rat fracture models, LIPUS can increase the expression of growth factors in the diabetic group and promote cartilage formation and angiogenesis (127). An experiment aimed at the effect of LIPUS on the alveolar structure in the process of orthodontic force in diabetic patients found that the application of LIPUS treatment, 10 minutes a day for one week, can promote normal and diabetic mandibular slice organ culture (MSOCs) bone remodeling and restoration of cementum and dentin (128). Although we believe that ultrasound has clinical potential in promoting bone healing in patients with diabetes and can even be used in the dental field, more experiments are still needed to prove and explore the underlying mechanism.

### Laser

Photobiomodulation (PBM) is a nonthermal light treatment that involves endogenous chromophores. Previous experiments have shown that PBM can promote bone healing in diabetic rat models by improving the viability of osteoblasts and mesenchymal cells (129, 130). Experiments on diabetic rats proved that PBM treatment can improve the viability of osteoblasts, significantly increase the mRNA expression of RUNX2 and osteocalcin, and increase the activity of alkaline phosphatase and the production of the mineralized matrix, thereby regulating the bone healing process (131, 132). In addition, PBM has also been shown to improve the survival, proliferation and apoptosis of BMSCs in diabetic rats (133).

Low-level laser therapy (LLLT) refers to radiation with a wavelength range of 500-1100 nm and a power of 1 mW-500 mW. It has the characteristics of relatively low energy density and has been used clinically to treat various diseases (134). The effect of LLLT in promoting bone healing has been verified by a large number of experiments, mainly by promoting the proliferation of osteoblasts, increasing the growth factor secreted by osteoblasts (135), the transportation of calcium (136) and increasing angiogenesis to promote bone healing (137). However, there are relatively few studies on diabetic bone healing. A previous study on the effect of LLLT and dual-type allograft materials on the healing of diabetic bone found that LLLT can effectively stimulate osteoblast production but cannot promote bone formation (138). However, other studies have shown that LLLT can stimulate bone metabolism, reduce bone resorption area, increase RUNX-2 expression, increase serum alkaline phosphatase levels, increase cortical area, fracture strength, BMD and bone mineral content (BMC), and promote bone healing (139, 140). Different experiments have shown almost the opposite results, which suggests that whether LLLT can promote the healing of diabetic bone, as well as the specific mechanism of promoting diabetic bone healing, still need many experiments to be studied.

### Pulsed Electromagnetic Field

In the past few decades, low-intensity pulsed electromagnetic fields (LIPEMFs) have played a positive role in the skeletal system. The application of PEMF treatment for patients with osteoporosis can significantly increase bone density and prevent bone loss (141, 142). Studies on animal models of diabetes found that LIPEMF can restore the expression of Runx2 through the Wnt/β-catenin signaling pathway and reverse the deterioration of bone microstructure and strength, thereby preventing bone loss caused by diabetes, but has no effect on osteoclasts (143–145). The positive effect of LIPEMF in promoting bone healing has been confirmed in a large number of experiments. Many preclinical experiments have proven that LIPEMF can effectively promote bone healing in diabetic animal models. However, there is still a lack of clinical trials for bone healing in diabetic patients. Although we believe that LIPEMF has clinical potential in promoting diabetic bone healing and may become a potential method to inhibit diabetic osteoporosis, its effectiveness lacks clinical experimental support.

To date, with the continuous progress of preclinical studies, the mechanism of HBO therapy, LIPUS, PBM, LLLT and LIPEMF in promoting diabetic bone healing has been gradually clarified. We believe that the physical therapies mentioned above have clinical potential in promoting diabetic bone healing. However, there have been no clinical studies to confirm these findings. With the gradual increase in the number of patients with diabetes and the occurrence of a large number of related bone healing complications, we believe that it is necessary to further study the potential role and safety of physical replacement therapy in bone healing in patients with diabetes, and these therapies may become an important part of the treatment of bone healing complications of diabetes.

## Challenges and Prospects

**Figure 2:**
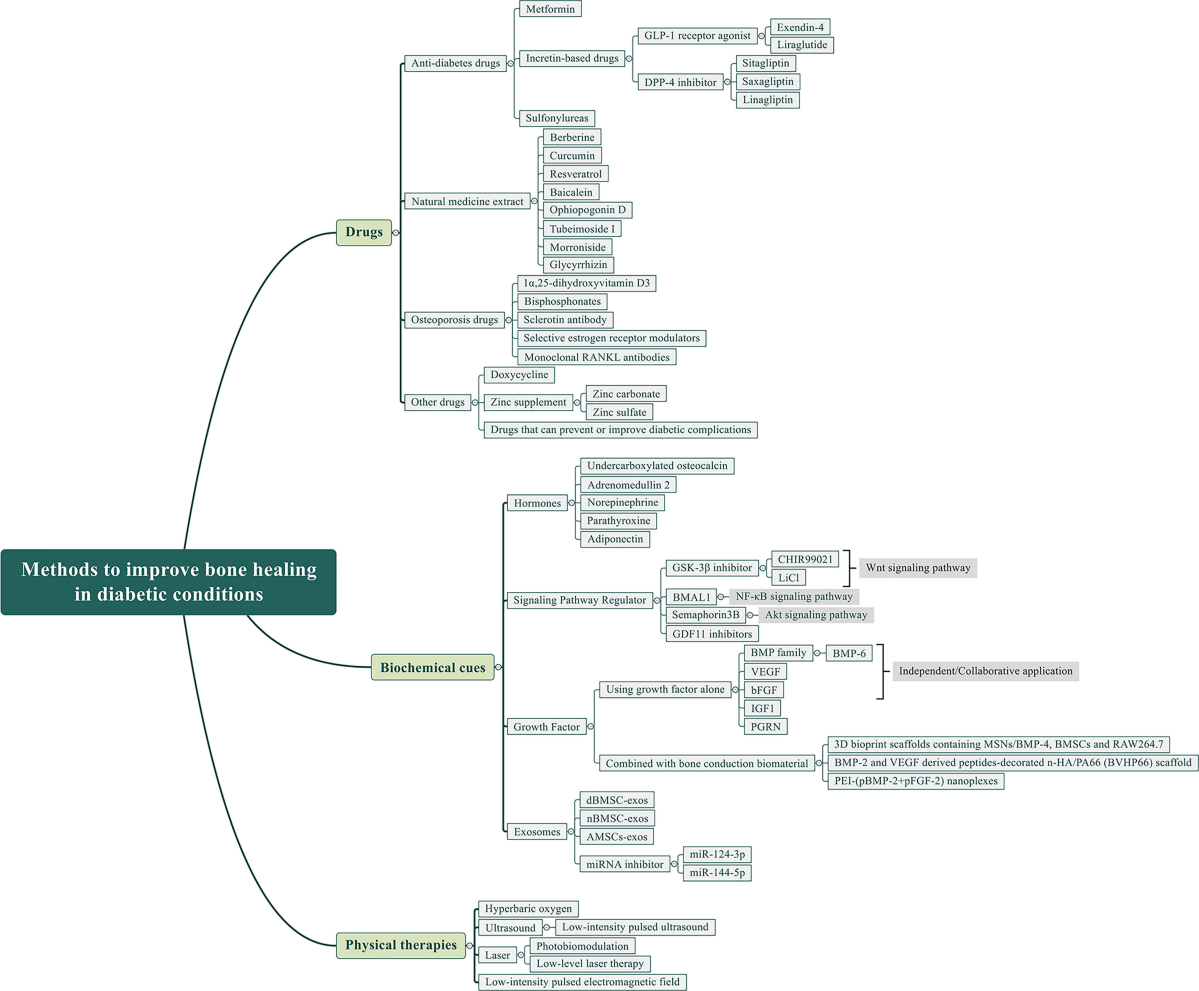
Methods to improve bone healing in diabetic conditions.

With the improvement of living standards and the rising prevalence of diabetes, it is urgent to explore how to improve bone healing repair in diabetes. This paper reviews the effects and possible mechanisms of diabetes on bone healing and summarizes several current methods to improve bone healing under diabetic conditions, although some studies are still in their early stages, including the use of drugs, hormones, signaling pathway regulators, growth factors, exosomes, etc. In addition, some physical therapies, such as hyperbaric oxygen, ultrasound, laser, and pulsed electromagnetic fields, also have a certain clinical potential in promoting bone healing under diabetic conditions (**Figure 2**).

Some clinical trials have proven that drugs related to glycemic control are beneficial to bone health in patients with diabetes. For example, metformin can increase the level of bone formation marker in T2DM patients (146), liraglutide has an anti-resorptive effect on bone turnover in patients with T2DM (147), GLP-1 receptor agonists can increase BMD at multiple sites of the body in T2DM patients (148), denosumab can significantly increase BMD and decrease vertebral fracture risk in postmenopausal women with osteoporosis and diabetes (62), etc. LLLT can improve implant stability in patients with diabetes in 6 months trial (149). However, there is no clinical research evidence specifically designed to improve fracture healing in patients with diabetes (72). And at present, there is no direct proof in existing clinical trials that the abovementioned methods have beneficial effects in improving bone healing under diabetic conditions. Therefore, clinical research on the methods mentioned above still lacks research, and their effectiveness and potential for clinical application still need to be further explored. Whether they can be adopted by clinical applications also needs further discussion.

## Author Contributions

YC and YZ conceived and wrote the manuscript. JL and SZ reviewed and edited the manuscript. All authors contributed to the article and approved the submitted version.

## Funding

This work was supported by grants from the Crosswise Tasks of Sichuan University (Document No. 21H0441, Funding No. 00403055A1042).

## Conflict of Interest

The authors declare that the research was conducted in the absence of any commercial or financial relationships that could be construed as a potential conflict of interest.

## Publisher’s Note

All claims expressed in this article are solely those of the authors and do not necessarily represent those of their affiliated organizations, or those of the publisher, the editors and the reviewers. Any product that may be evaluated in this article, or claim that may be made by its manufacturer, is not guaranteed or endorsed by the publisher.
